# Evidence of Neutral Evolution of Mitochondrial DNA in Human Hepatocellular Carcinoma

**DOI:** 10.1093/gbe/evz214

**Published:** 2019-10-10

**Authors:** Qi Liu, Deng Lin, Mingkun Li, Zhenglong Gu, Yiqiang Zhao

**Affiliations:** 1 Beijing Advanced Innovation Center for Food Nutrition and Human Health, College of Biological Sciences, China Agricultural University, Beijing 100193, China; 2 State Key Laboratory of Agrobiotechnology, China Agricultural University, Beijing 100193, China; 3 Key Laboratory of Genomic and Precision Medicine, Beijing Institute of Genomics, Chinese Academy of Sciences, Beijing, China; 4 Center for Excellence in Animal Evolution and Genetics, Chinese Academy of Sciences, Kunming, China; 5 Division of Nutritional Sciences, Cornell University, Ithaca, New York 14853

**Keywords:** mitochondrial DNA, heteroplasmy, neutral selection, hepatocellular carcinoma (HCC)

## Abstract

Many studies have suggested that mitochondria and mitochondrial DNA (mtDNA) might be functionally associated with tumor genesis and development. Although the heterogeneity of tumors is well known, most studies were based on the analysis of a single tumor sample. The extent of mtDNA diversity in the same tumor is unclear, as is whether the diversity is influenced by selection pressure. Here, we analyzed the whole exon data from 1 nontumor sample and 23 tumor samples from different locations of one single tumor tissue from a hepatocellular carcinoma (HCC) patient. Among 18 heteroplasmic sites identified in the tumor, only 2 heteroplasmies were shared among all tumor samples. By investigating the correlations between the occurrence and frequency of heteroplasmy (Het) and sampling locations (Coordinate), relative mitochondrial copy numbers, and single-nucleotide variants in the nuclear genome, we found that the Coordinate was significantly correlated with Het, suggesting no strong purifying selection or positive selection acted on the mtDNA in HCC. By further investigating the allele frequency and proportion of nonsynonymous mutations in the tumor mtDNA, we found that mtDNA in HCC did not undergo extra selection compared with mtDNA in the adjacent nontumor tissue, and they both likely evolved under neutral selection.

## Introduction

Mitochondria play a pivotal role in eukaryotic cells. Oxidative phosphorylation reactions that occur in mitochondria supply the energy needed for cellular processes. Mitochondria are also involved in other important functions, including cell signaling and cell death, and they are the key component in innate immunity (West et al. 2015; Van Houten et al. 2016). Mitochondria have their own genetic materials named mitochondrial DNA (mtDNA), which is characterized as multicopy, lacking genetic recombination, and prone to mutations. The number of mitochondria per cell varies from a few hundred to tens of thousands in different cell types. The coexistence of wild-type and mutant mtDNA in the same cell is called mitochondrial heteroplasmy. MtDNA mutations have been reported to be associated with multiple diseases, including Leber’s hereditary optic neuropathy, deafness, autism, and Parkinson’s disease, as well as tumors, and these mutations always occur in the heteroplasmic state (Wallace et al. 1999; Farrar et al. 2013).

Mutations in mtDNA (Zhai et al. 2011; Gutiérrez Povedano et al. 2015; Guo et al. 2016; Chen et al. 2017; Triska et al. 2019) and nuclear-encoded mitochondrial genes (Dang et al. 2010; Losman and Kaelin 2013; Gaude and Frezza 2014) were associated with cancers. Based on the analysis of 188 hepatocellular carcinoma (HCC) patients and 344 healthy individuals, Chen et al. (2017) proposed that patients with haplogroups M7 and M8 had relatively higher risk of developing HCC (Bussard and Siracusa 2017). In another study, Er et al. (2017) reported that fewer T489C (mutation from T to C at position 489) and T16519C mutations were observed in gastroenteropancreatic–neuroendocrine neoplasms patients compared with healthy controls. MtDNA mutations promote tumorigenesis presumably because some mutations lead to overproduction of reactive oxygen species, which in turn contribute to the activation of the oncogenic signaling (Ishikawa et al. 2008; Liou and Storz 2010).

A phenomenon called the Warburg effect should be considered when discussing the role of mitochondria in carcinogenesis. The Warburg effect refers to the tendency that cancer cells have to rely on aerobic glycolysis to generate energy rather than the more efficient oxidative phosphorylation (Warburg 1956), where the latter occurs in the membranes of mitochondria. However, this does not mean that mitochondria are dispensable for the cancer cell; recent studies showed a considerable delay in tumorigenesis for cancer cells without mtDNA (ρ^0^ cells) (Tan et al. 2015; Dong et al. 2017).

Studies suggested that purifying selection could exert an effect on mtDNA mutations (Elson et al. 2004; Pereira et al. 2011; Avital et al. 2012; Li, Rothwell, et al. 2016), and the selection could be even stronger during the process of growth and aging (Li et al. 2010; Freyer et al. 2012). In contrast, other studies proposed that mtDNA mutations in tumors seem to occur under neutral evolution (Ju et al. 2014; Grandhi et al. 2017). Ericson et al. (2012) observed a lower number of mtDNA mutations in human colorectal cancer tissues, in favor of a neutral model, and argued that the decreased mtDNA mutagenesis observed in the tumor was a byproduct of reduction of reactive oxygen species in the tumor.

Besides mutations, alterations in mtDNA copy number (mtCN) have been observed in various cancer types. However, different studies showed conflicting results. Decreases in mtCN were reported in breast cancer, liver cancer, and clear-cell kidney cancers, whereas mtCN was reported to be increased in lung adenocarcinoma (Reznik et al. 2016). A meta-analysis by Hu et al. (2016) showed that although, as a whole, no significant association exists between mtCN and cancer risk, they observed a positive association between mtCN and lymphoma and breast cancer, but a negative association between mtCN and hepatic carcinoma. Even for the same cancer, the results could be inconsistent (Mambo et al. 2005; Reznik et al. 2016).

Previous studies on the tumor mitochondrial genome were mainly performed on individual samples. Considering the genetic materials in the tumor being highly variable (Li et al. 2018), the results could be influenced by the sampling effect. The comparison of mtDNA from different parts of the same tumor could shed light on the selection pattern on the mtDNA in the tumor, as the mtDNA should be more diverse in the absence of selection. Ling et al. (2015) investigated the intratumor genetic diversity by employing whole-exome sequencing on samples from 23 different locations on one HCC tissue. They found that somatic mutations in HCC exhibited a pattern of non-Darwinian evolution, and mutations at different locations of the same tumor evolved under a neutral model.

In this study, we reanalyzed the exon data and focused on the mtDNA variations. We found that the distribution and frequency of mtDNA mutations were strongly correlated with their spatial locations and adjacent samples tended to share the same mtDNA mutations. Similar Ka/Ks ratios (not different from one) and minor allele frequency (MAF) distributions were observed in tumor and adjacent nontumor tissues, suggesting that mtDNA in the HCC exhibit a pattern of neutral evolution.

## Materials and Methods

### Exon Data for Hepatic Carcinoma

The exon sequencing data used in this study were obtained from the study of Ling et al. (2015) (which is hereinafter referred to as the original study). In the original study, 23 samples were collected from a ∼35 mm in diameter region of single tumor tissue, and 1 adjacent nontumor sample was also collected. The average sequencing depth was 70×. Raw sequencing files were downloaded from Genome Sequence Archive, Beijing Institute of Genomics (http://gsa.big.ac.cn accession no. PRJCA000091; last accessed October 09, 2019).

### MtDNA Mutation Detection

Two types of mutations (substitution and heteroplasmy) were detected and analyzed in the study. We first mapped all whole-exome sequencing reads to the GRCh38 reference genome, including the revised Cambridge Reference Sequence (rCRS) (NC_012920) using BWA mem (Li and Durbin 2009) with default settings. All read pairs perfectly aligned with the nuclear genome were removed to exclude nuclear mitochondrial DNA segments (NUMTs). We then created a pseudo mitochondrial reference sequence by copying the first 500 bp of rCRS to its end, thus producing a pseudo rCRS of 17,069 bp, which could significantly improve the mapping quality at the D-loop region. The filtered reads were then mapped to the pseudo rCRS using BWA mem. Samtools (Li et al. 2009) was used to process the bam files to retrieve read pairs that were perfectly aligned with the pseudo rCRS, and reads with a flag “SECONDARY” or “SUPPLEMENTARY” were filtered out. After filtering, Samtools mpileup function was used to create the pileup file containing 17,069 sites, with a minimum base quality score of 30. We finally converted the 17,069 sites to 16,569 sites by pileuping the reads mapped to the overlap region. A substitution was identified if two criteria were met: 1) the major allele on the forward and reverse strands were same, but different from the reference allele; and 2) the major allele was supported by more than three reads on both strands. Heteroplasmy was reported if four criteria were met: 1) the major and minor alleles on the forward and reverse strands were same; 2) the major and minor alleles were supported by more than three reads on both strands; 3) the frequency of the minor allele was significant >1% by considering a binomial distribution; and 4) the positions were not in low-complexity regions and ambiguous regions (302–316, 513–526, 566–573, 3,106–3,107, and 16,181–16,194) (Zheng et al. 2011; Rebolledo-Jaramillo et al. 2014; Li et al. 2015).

The aforementioned method was implemented in an R package named MitoMutCall and uploaded to github (https://github.com/apiaolin/MitoMutCall; last accessed October 09, 2019). To evaluate the precision and recall rate of the method, simulated reads were generated by Wgsim using the rCRS sequence as the reference genome, the sequencing error rate was set to 3‰ and the read length to 100 bp (Li et al. 2009). The MAF of the heteroplasmies were set to 1.5%, 2.5%, 3.5%, 4.5%, 5.5%, 6.5%, 7.5%, 8.5%, 9.5%, 10.5%, and 20.5%. For each condition, the number of reads was set to 100,000, 200,000, 400,000, 600,000, 800,000, 1,000,000 to simulate different sequencing depths (∼500×, 1,000×, 2,000×, 3,000×, 4,000×, and 5,000×, respectively). For each setting, the simulation was repeated 100 times.

### Prediction of the Deleteriousness of Mutations

CADD v1.3 (Kircher et al. 2014) was used to annotate mutations. According to the CADD manual, mutations with C-score >15 were considered as potentially pathogenic.

### Calculation of Relative mtDNA Copy Number

We used the method proposed by Reznik et al. (2016) to calculate the mtCN in tumors. Briefly, a depth ratio was calculated by dividing the mtDNA sequencing depth (mt_depth) by the CDS sequencing depth (CDS_depth). The mtCN was then calculated by multiplying the depth ratio by the ploidy of the nuclear genome (eq. 1). However, due to the existence of gene duplication and deletion events occurring in tumor cells, the ploidy was not two. The correct ploidy number, *R*_Tumor_, was calculated using equation (2). CDS_depth, Purity, and Ploidy were retrieved from the Supplementary Material online of the original study (Ling et al. 2015).

(1) $$\text{mtCN}=\frac{\text{mt}\_\text{depth}}{\text{CDS}\_\text{depth}}\times R_{\mathrm{Tumor}}$$

(2) $$R_{\mathrm{Tumor}}=\frac{\mathrm{Purity}\times \mathrm{Ploidy}+(1-\mathrm{Purity})\times 2}{2}$$

### Dendrogram Construction

Due to data availability, we considered four characteristics in this study: the Coordinate, Het, mtCN, and single-nucleotide variants (SNV). For Coordinate, we set up a coordinate system according to figure 2*A* in the original study. Because Z1 was in the middle of the sliced piece of the tumor, it was chosen to be the origin of the Cartesian coordinate system. Coordinates of each sample are listed in the supplementary table S1, Supplementary Material online. The pairwise Euclidean distance among all samples was calculated and saved in a matrix. A Het matrix was generated based on the differences between minor allele frequency (MAF) of all heteroplasmy sites (supplementary table S2, Supplementary Material online). Similarly, the mtCN distance matrix was calculated based on the differences between mtCN (supplementary table S3, Supplementary Material online). For SNV, we used the matrix constructed in the original study (supplementary table S4, Supplementary Material online). Four matrices were finally normalized by scaling the value between 0 and 1. The “APE” R package (Paradis et al. 2004) and iTOL (itol.embl.de; last accessed October 09, 2019) were used for constructing and plotting the dendrogram.

### Calculation of Distance between Different Dendrograms

We used R package “phangorn” (Schliep 2011) to calculate the weighted Robinson–Foulds distance (wRFD) between the four dendrograms constructed in the previous step. The statistical significance of the wRFD was assessed by randomly assigning the labels of the leaf nodes on the dendrogram for 1,000 repeats. Finally, the dendrograms built from the four characteristics were converted into one single distance matrix presenting the interrelationships among Coordinate, Het, mtCN, and SNV.

### Analysis of the Nuclear-Encoded Mitochondrial Genes

We downloaded a list of 1,145 nuclear-encoded mitochondrial genes from MitoCarta2.0 (Calvo et al. 2016). By searching against all SNVs identified in the tumor and the nontumor tissues (supplementary data set S2, Supplementary Material online, in the original study), we found 12 SNVs located in the nuclear-encoded mitochondrial genes, and 257 SNVs were located in other nuclear genes. The mutation frequency and the ratio of nonsynonymous to synonymous mutations were calculated and compared between nuclear-encoded mitochondrial genes and other nuclear genes.

### Ka/Ks Calculation

MtDNA mutations located in coding regions in all tumor samples were coalesced into a single sequence. Codeml program in PAML (Yang 1997) was used to calculate the Ka/Ks value for both nontumor and tumor tissues. The 95% confidence intervals (CIs) were calculated by permutation tests (mutations were randomly sampled 1,000 times).

## Results

### Accurate and Rapid Detection of mtDNA Mutations

An algorithm (MitoMutCall) was developed for the detection of heteroplasmy, which only required an average of ∼13 seconds (10.76–22.17 s) to report mtDNA mutations starting from pileup files (with a 20-thread CPU). The precision and recall of the method were first evaluated using a simulated data set, the precision was >0.99 even for the lowest frequency mutation (1.5%) when the sequencing depth was 500, whereas the mean recall was >0.9 for the mutation with frequency of 1.5%, and close to one for higher frequency mutations (supplementary tables S6 and S7, Supplementary Material online), suggesting the method had a good sensitivity and specificity for detecting low-frequency heteroplasmies. We further compared our results (105 heteroplasmy sites) with those obtained with the DREEP method (89 heteroplasmy sites) (Li and Stoneking 2012), which applied a similar algorithm but was slower (>1 min), We found that all heteroplasmic sites identified by DREEP were reported by our method, but not vice versa. We further confirmed that the discrepancy was caused by a more stringent filter on the alignment mismatch number applied in DREEP (two mismatches were allowed by default), which led to the discard of some extra reads. Since the length of reads was significantly longer than that generated before (150 bp vs. 76 bp), we thought two mismatches was too stringent; thus, the mismatch number was not used to filter the result by MitoMutCall. An R package implementing this algorithm was uploaded to github (https://github.com/apiaolin/MitoMutCall; last accessed October 09, 2019).

### Differences in mtDNA between Tumor and Nontumor Samples

Compared with the N1 mtDNA sequence, one substitution T16117C was identified in all tumor samples, suggesting this substitution probably occurred in the primitive tumor cell, thus was inherited by all progeny tumor cells. No other substitution was identified in tumor samples. Position 16117 was in the D-loop region and close to the mtDNA replication origin Ori-b site, so we suspected that the mutation might affect the replication of mtDNA. The deleteriousness score of the substitution was 10.04, estimated by CADD (Kircher et al. 2014), which was below the proposed deleterious threshold. The substitution was not associated with any disease in the Mitomap database.

We detected 105 heteroplasmies in 24 samples (supplementary table S2, Supplementary Material online), which were located at 31 positions. We identified 14 heteroplasmic sites in N1, which was higher than in tumor samples (2–7 with an average number of 3.96). Unexpectedly, 13 of them were specific to N1; as the patient was infected with Hepatitis B Virus (HBV) and liver cirrhosis, this sample may not be an appropriate sample to represent the health status, but instead could be an independent clone used as an outgroup in the analysis of mtDNA in the tumor. Since the size of the nontumor tissue was much larger than that of each tumor microsections (the size of the tumor and nontumor tissues were both 35 × 35 × 10 mm), it was unclear how many cells were sequenced for the nontumor tissue; the sample size may have been larger for the nontumor tissue, in which case, the number of heteroplasmies was the sum of different microsections. Seven heteroplasmic sites (out of 18) were observed in multiple tumor samples but with different frequencies; among them, two heteroplasmies (G14804A and T16117C) were observed in all tumor samples, and one heteroplasmy (G3424) was observed in 19 tumor samples (table 1). Notably, G3424A and G14804A were nonsynonymous mutations located in *ND1* and *CYB* gene, and their CADD scores were 23.20 and 19.05, respectively, suggesting the gene functions were likely affected. All the other heteroplasmies were only detected in a small number of tumor samples (<6) and 11 of them were observed in single tumor sample. We found no correlation between the heteroplasmy number with sequencing depth, sample purity, or mtCN (*P* value > 0.05).

**Table 1:** Summary of the Three Shared Heteroplasmic Sites in 23 Tumor Samples

Sample	Position 3424	Position 14804	Position 16117	HeterNum
A25	G/A: 0.0542	G/A: 0.1495	C/T: 0.0916	3
A5	G/A: 0.0787	G/A: 0.2037	C/T: 0.0180	3
A58	G/A: 0.0311	G/A: 0.2303	C/T: 0.0490	3
A61		G/A: 0.0566	C/T: 0.0332	2
A66	G/A: 0.0423	G/A: 0.0658	C/T: 0.1262	5
B33	G/A: 0.0135	G/A: 0.1395	C/T: 0.0371	3
B4	G/A: 0.0217	G/A: 0.2483	C/T: 0.0565	3
B45		G/A: 0.1536	C/T: 0.0735	3
B5	G/A: 0.0344	G/A: 0.3147	C/T: 0.0315	3
B6	G/A: 0.0243	G/A: 0.2854	C/T: 0.0783	3
B9	G/A: 0.0197	G/A: 0.1047	C/T: 0.0334	3
C2	G/A: 0.0508	G/A: 0.0251	C/T: 0.0213	4
C3	G/A: 0.0139	G/A: 0.0545	C/T: 0.0431	7
C31	G/A: 0.0246	G/A: 0.0290	C/T: 0.0399	7
C74	G/A: 0.0240	G/A: 0.0359	C/T: 0.0404	3
D16		G/A: 0.1282	C/T: 0.0243	3
D25	G/A: 0.0959	G/A: 0.0984	C/T: 0.0346	3
D29	G/A: 0.0271	G/A: 0.1319	C/T: 0.0241	4
D54	G/A: 0.0169	G/A: 0.0993	C/T: 0.0166	4
D58	G/A: 0.0264	G/A: 0.1678	C/T: 0.0258	5
D62	G/A: 0.0573	G/A: 0.1297	C/T: 0.0344	6
D63	G/A: 0.0319	G/A: 0.1403	C/T: 0.0445	5
Z1		G/A: 0.0232	C/T: 0.0296	6

Note.—Major allele, minor allele, and minor allele frequency were shown in the table. For example, G/A: 0.0542 means that the major allele at this position is G while the minor allele is A, and the minor allele frequency is 0.0542. HeterNum represents the total number of heteroplasmies identified in the sample.

### MtDNA Heteroplasmies Shared among Adjacent Samples

**Fig. 1.**
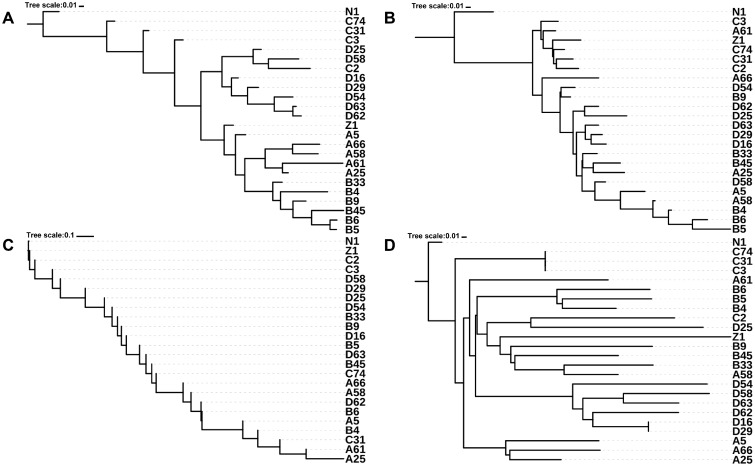
—Dendrograms constructed based on different distance matrices. (*A*) Coordinate, (*B*) Het, (*C*) mtCN, and (*D*) SNV dendrogram. Note that (*C*) was created from scalar values of the copy number; thus, the tree cannot branch.

By setting up a coordinate system, the pairwise physical distances for all sampling locations were measured and a dendrogram was constructed (fig. 1*A*). N1 was set to be the root of the dendrogram as it was the only nontumor sample. Another dendrogram was built according to the shareness of heteroplasmies (fig. 1*B*). We noticed that samples from adjacent sites tended to be closer in the heteroplasmy dendrogram, which was especially evident for two subgroups (B4, B5, and B6 and D16, D29, and D63). Similarly, we obtained the dendrograms of mtCN and nuclear SNV (fig. 1*C* and *D*).

**Fig. 2.**
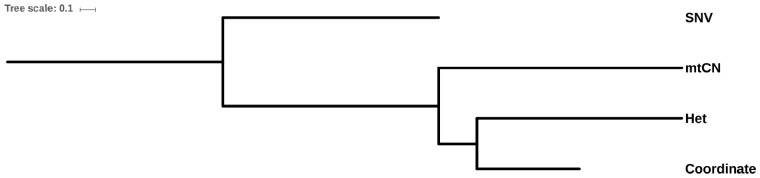
—The interrelationship between the four characteristics. Dendrograms were constructed based on the wRFD matrices.

The similarities among these dendrograms were further measured using the wRFD (Robinson and Foulds 1981). The significance of the similarity was evaluated using a permutation test. We found that the Coordinate dendrogram was significantly similar to the Het dendrogram (wRFD = 2.70; *P* value < 0.001), but less similar when compared with the mtCN dendrogram and SNV dendrogram (table 2 and fig. 2; *P* value > 0.05). The correlation between the occurrence and frequency of heteroplasmy and the spatial location suggests that mtDNA mutations occurred during the development of HCC under no strong selective constraints or positive selection. No significant correlation was found between the occurrence of mtDNA mutations, nuclear mutations, and the number of mtCNs, suggesting they were independent events during the development of tumor cells. We found that the greatest distance between tumor cells and nontumor cells was observed in the Het dendrogram, implying that Het could be involved in the development of HCC. However, this could also be associated with the specificity of the nontumor mtDNA used in the study (HBV-infected and liver cirrhosis).

**Table 2:** wRFD Distances between Four Dendrograms

	Coordinate	Het	SNV	mtCN
Coordinate	0	—	—	—
Het	2.70***	0	—	—
SNV	5.73	5.17	0	—
mtCN	3.21	3.19	6.13	0

Note.—The similarity between two dendrograms was assessed by randomly assigning the labels of the leaf nodes on the dendrogram for 1,000 times. Dendrogram pairs that with significantly smaller distances (*P* value < 0.001) are labeled with three asterisks.

### No Strong Selective Pressure Observed on mtDNA Mutations in HCC Tumor Cells

**Fig. 3.**
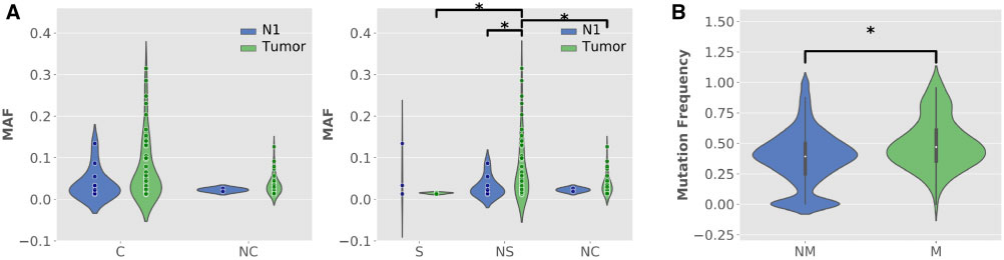
—Comparison of the minor allele frequency (MAF) in different mutation categories. (*A*) Comparison between N1 and tumor samples (tumor), C denotes the coding region, NC denotes the noncoding region, S denotes synonymous mutations, and NS denotes nonsynonymous mutations. (*B*) Comparison of the frequency of SNV in nuclear-encoded mitochondrial genes (M) and other nuclear genes (NM). Mann–Whitney *U* test was used to calculate the significance of differences. Asterisk was added to denote a significant difference (*P* value < 0.05).

To further verify the neutral evolution of mtDNA in HCC, we investigated the heteroplasmy sites with different degrees of functional importance. The heteroplasmies were classified into different categories by their locations and the nature of the mutations. They were first classified as coding (C) and noncoding (NC) heteroplasmies, and the former was further classified as nonsynonymous (NS) and synonymous (S) heteroplasmies. In total, there were in total 12 C heteroplasmies (3 S heteroplasmies and 9 NS heteroplasmies) and 2 NC heteroplasmies in the N1 sample. In contrast, 59 C heteroplasmies (4 S heteroplasmies and 55 NS heteroplasmies) and 32 NC heteroplasmies were identified in all tumor samples. As the same heteroplasmy detected in multiple tumor samples was likely caused by a single mutation event, such heteroplasmies were counted once in calculating the Ka/Ks ratio, which were 0.59 (0.68–0.76, 95% CI) and 1.17 (1.02–8.13) for the tumor and nontumor tissues, respectively. The Ka/Ks ratio was not significantly different between the tumor and nontumor tissues (*P* value > 0.05) and neither of the ratios significantly differed from one (pvalue > 0.05). The MAF of the heteroplasmies in different categories was similar in both tumor and nontumor tissues, except the NS heteroplasmies in tumors had a higher MAF (fig. 3*A*). The results indicate that no pervasive purifying selection acted on mtDNA in HCC, whereas positive selection was possible on some nonsynonymous mutation.

As most evidence supported a neutral selection or a moderate level of purifying selection acting on mtDNA in HCC, we explored whether nuclear-encoded mitochondrial genes were also under weaker purifying selection than nuclear genes, as most nuclear genes evolved under purifying selection (Hamosh et al. 2004; The Genomes Project Consortium et al. 2012). We identified 269 nuclear SNVs in 23 tumor samples. Among these SNVs, 12 SNVs were located in nuclear-encoded mitochondrial genes (supplementary table S5, Supplementary Material online), including 9 nonsynonymous mutations and 3 synonymous mutations. The proportion of nonsynonymous mutations was similar to that of the other nuclear genes (199 nonsynonymous mutations and 44 synonymous mutations, *P* value > 0.05). The mutation frequency of SNVs in nuclear-encoded mitochondrial genes was higher than that in other nuclear genes (*P* value = 3.51e-15, fig. 3*B*), suggesting that nuclear-encoded mitochondrial genes might experience weaker purifying selection compared with other nuclear genes.

Taken together, our results suggest that mtDNA does not evolve under strong purifying selection in HCC patients. No signal of strong positive selection was detected; thus, overall, a nearly neutral evolution model was favored by the data.

## Discussion

Due to the nature of higher mutation rate and multiple copies of mtDNA in a single cell, intraindividual mutations were not rare in mtDNA. However, most of these mutations had a low frequency and were hard to fix in the individual. A frequency threshold was thought to exist (Rossignol et al. 2003), above which the function of mitochondria cannot be retained; thus, a purifying selection on mtDNA has been widely accepted (Stewart et al. 2008). The situation is complicated in cancer cells. Cancer cells favor aerobic glycolysis instead of oxidative phosphorylation, or the Warburg effect, which predicts that tumor cells might be able to tolerate impaired mitochondrial functioning (Warburg 1956).

Previous studies reported that mutations in D-loop region were associated with HCC (Lee et al. 2004; Yin et al. 2004, 2010; Li, Wan, et al. 2016). Other studies reported that the mtDNA copy number in liver cancer was decreased compared with normal tissues and was negatively correlated with hepatic malignancy (Lee et al. 2004; Yamada et al. 2006; Yu et al. 2018), suggesting that the change in mtDNA is associated with the development and prognosis of diseases, whereas the mitochondrial function in liver cancer cells might not be well retained. However, whether the mtDNA in disease (especially in tumors) is under strong selection pressure is still arguable.

Unlike previous studies that were based on the comparison of mtDNA mutations between tumors and normal tissues, we attempted to trace the development and change in mtDNA mutations in different locations of the same tumor. The low level of shared mutations (3 out of 18) among different locations and the significant correlation between mutations and spatial distribution, suggest that no strong purifying selection or positive selection acts on mtDNA in HCC. By further considering the results of Ka/Ks (not significantly differing from one) and MAF (not differing much between mutations with different importance) analysis, we speculate that the evolution of mtDNA in the tumor was more likely to be neutral. However, our data cannot rule out the possibility of a relaxed purifying selection or a weak positive selection. More complicated scenarios, like balanced positive and purifying selections or existence of evolutionary constrains (e.g., hitchhiking) that impair selective pressure, are also possible.

The major limitation of our study is that all conclusions were based on the analysis of samples from a single individual (as only one intratumor data set was available when the study was conducted). To determine if the scenario is similar in other individuals or for other tumors, further studies are needed to confirm our findings in a larger data set. Another limitation is the single and nonstrict control used in the study, which prevents distinguishing real tumor-specific heteroplasmies from background heteroplasmies; however, our conclusions are unlikely to be influenced by this issue as the dendrogram was created on the occurrence and frequency change of mutations. Nontumor-specific mutations were as informative as tumor-specific mutations in this analysis. 

## Supplementary Material

Supplementary data are available at *Genome Biology and Evolution* online.

## Supplementary Material

evz214_Supplementary_Data
